# Cutaneous Mast Cell Tumor With Metastasis in a Pet Cairo Spiny Mouse (*Acomys cahirinus*): A Case Report With Clinicopathologic Characterization

**DOI:** 10.1155/crve/5401840

**Published:** 2026-09-19

**Authors:** Stephanie E. Anderson, Amanda Smith, Shanna R. Nelson, Keiichi Kuroki

**Affiliations:** ^1^ Veterinary Medical Diagnostic Laboratory, Department of Pathobiology and Integrative Biomedical Sciences, College of Veterinary Medicine, University of Missouri, Columbia, Missouri, USA, umkc.edu; ^2^ Fox Creek Veterinary Hospital, Wildwood, Missouri, USA

**Keywords:** mast cell tumor, metachromasia, metastasis, spiny mouse

## Abstract

A 3‐year‐old male pet Cario spiny mouse (*Acomys cahirinus*) presented with an exophytic, sessile dermal mass at the base of the left ear. Histopathological examination of the excisional biopsy revealed a poorly demarcated, densely cellular neoplasm composed of sheets of round to polygonal cells containing variably distinct basophilic to amphophilic fine intracytoplasmic granules. The granules exhibited metachromasia with toluidine blue and Giemsa stains. Neoplastic cells were immunopositive for c‐kit (CD117) and tryptase, confirming a diagnosis of mast cell tumor (MCT). The tumor was narrowly excised, and the mouse remained clinically well 1 month postsurgery without evidence of recurrence; however, the mouse declined clinically with tumor recurrence and was euthanized at 3 months postsurgery. Necropsy revealed local recurrence with metastasis to regional lymph nodes and spleen. This case report represents, to the authors′ knowledge, the first report of a cutaneous MCT in a Cario spiny mouse and demonstrates malignant biological behavior. This report highlights the importance of considering MCTs in the differential diagnosis of cutaneous masses in exotic rodents.

## 1. Introduction

Mast cell tumors (MCTs) are among the most common cutaneous neoplasms in dogs and cats, accounting for approximately 20% of all skin tumors [1, 2]. In contrast, MCTs are seldom reported in mice, with most reported MCTs occurring in laboratory settings and often associated with experimental radiation or chemical induction, with few spontaneous cases documented [3–6]. MCTs in mice have been described as soft, white nodules with densely packed uniform round cells arranged in rows or clusters with distinct cell borders, abundant cytoplasm, and a central nucleolus [7]. The cytoplasm appears faintly granular with eosinophilic to amphophilic granules displaying metachromasia on Giemsa and toluidine blue [7]. Reported tumor locations in mice include skin, forestomach, bone marrow, spleen, lungs, and liver, with evidence of dissemination in four cases [3, 5, 6]. Although one MCT case was reported in a pet African dormouse (*Graphiurus* sp.) [8], most published murine cases are laboratory mice, suggesting that MCTs may be underdiagnosed in pet mice. MCTs have also been infrequently reported in other rodents such as rats [3, 9, 10], a ground squirrel [11], hamsters [12], and Mongolian gerbils [13]. As exotic rodents become increasingly common in veterinary practice, clinicians and pathologists may encounter neoplastic conditions that are poorly characterized in these species. This report describes the clinicopathologic features and biological behavior of a cutaneous MCT in a pet Cairo spiny mouse (*Acomys cahirinus*), emphasizing diagnostic features and clinical relevance.

## 2. Case Description

**Figure 1:**
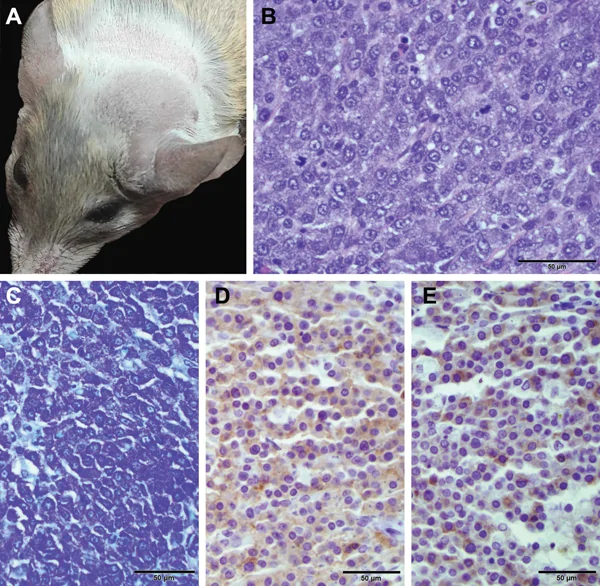
Mast cell tumor in a Cairo spiny mouse. (A) The base of the left ear is expanded by a poorly demarcated, soft, exophytic, sessile dermal mass measuring approximately 1.5 × 1.0 × 0.5 cm. (B) Round to polygonal neoplastic cells are arranged in sheets containing ample amphophilic cytoplasm with variably distinct basophilic to amphophilic granules with variably distinct cell borders. Nuclei are round with vesicular to marginally condensed chromatin and one to two distinct nucleoli. Anisocytosis is moderate, and two mitotic figures are observed in 2.37 mm^2^. Eosinophils are seen scattered throughout neoplastic cells. H&E bar, 50 μm. (C) The intracytoplasmic granules stain positively with toluidine blue. Bar, 50 μm. (D) Neoplastic cells show diffuse cytoplasmic immunopositivity for c‐kit. Bar, 50 μm. (E) Neoplastic cells show diffuse cytoplasmic immunopositivity for tryptase. Bar, 50 μm.

A 3‐year‐old male pet Cairo spiny mouse presented with a rapidly developed, poorly demarcated, exophytic, sessile dermal mass (1.5 × 1.0 × 0.5 cm) at the base of the left ear (Figure 1A). The lesion was initially suspected to be a bacterial infection but did not respond to antibiotic therapy. Surgical excision, including the left pinna, was performed for both therapeutic and diagnostic purposes.

### 2.1. Histopathology

The biopsy specimen was fixed in 10% neutral buffered formalin and submitted to the University of Missouri′s Veterinary Medical Diagnostic Lab (VMDL) for histopathologic examination. After routine histologic processing, 5‐*μ*m‐thick sections were stained with hematoxylin and eosin (H&E) for routine histopathological evaluation. Histopathologic examination revealed an unencapsulated, poorly demarcated, highly cellular neoplasm expanding the dermis and extending into the subcutis. The neoplasm consisted of sheets of round to polygonal cells with variably distinct cell borders and ample pale amphophilic cytoplasm containing variably distinct fine basophilic to amphophilic granules. Nuclei were round to oval with vesicular to marginally condensed chromatin and one to two variably distinct nucleoli (Figure 1B). Anisocytosis and anisokaryosis were moderate, with a low mitotic count (2 in 2.37 mm^2^). Scattered small numbers of eosinophils were observed throughout the neoplasm. The neoplasm was narrowly excised, with neoplastic cells extending to within < 1 mm of the deep surgical margin.

### 2.2. Histochemical and Immunohistochemical Investigation

Histologically, a cutaneous MCT was suspected and further evaluated using toluidine blue and Giemsa histochemical stains, along with immunohistochemistry for c‐kit (CD117) (1:400 dilution, A4502, DAKO) and tryptase (1:100 dilution, M7052, DAKO). The intracytoplasmic granules of neoplastic cells exhibited metachromasia with toluidine blue (Figure 1C) and Giemsa (not shown). Immunohistochemically, the neoplastic cells showed diffuse cytoplasmic positivity for c‐kit (Figure 1D) and tryptase (Figure 1E). These findings confirmed the diagnosis of an MCT.

### 2.3. Clinical Outcome

The mouse recovered uneventfully from surgery and remained clinically well 1 month postoperatively. However, at a 3‐month checkup, the mouse had seromas at the surgical site with the development of severe erosion and ulceration of the skin extending from the surgical site to surrounding the base of the right ear, concerning for tumor recurrence. Due to poor quality of life, euthanasia was elected, and the body was submitted for necropsy.

### 2.4. Necropsy Findings

**Figure 2:**
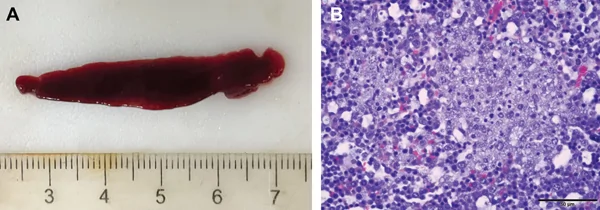
Mast cell tumor metastasis in the spleen in a Cairo spiny mouse. (A) The spleen is diffusely enlarged, measuring 4.6 × 0.9 × 0.1 cm. (B) There are multifocal metastatic clusters of the previously described neoplastic mast cells within the splenic parenchyma. H&E bar, 50 μm.

Postmortem examination revealed severe erosion and ulceration at the surgical site and adjacent tissues extending from the middle of the head and muzzle to the base of the shoulders, measuring 3 × 2.5 cm in diameter with a ~0.5 cm focal depressed pocket in the soft tissue. There was a second 1‐cm diameter pocket near the left shoulder that did not connect to the underlying tissue. A 0.5 × 0.1 cm piece of soft tan tissue was in the region of the right salivary gland, and the left and right submandibular lymph nodes were prominent. The spleen measured 4.6 × 0.9 × 0.1 cm and was subjectively enlarged with mildly rounded edges (Figure 2A). The remainder of the gross examination was unremarkable.

On microscopic examination, the skin at and adjacent to the surgical site was infiltrated by neoplastic round to polygonal cells similar to the neoplastic cells described in the original biopsy. Anisocytosis and anisokaryosis of the neoplastic cells were moderate, with five mitoses in 2.37 mm^2^. The intracytoplasmic granules of neoplastic cells exhibit metachromasia with toluidine blue, consistent with neoplastic mast cells and local tumor recurrence. Metastatic MCT was identified in submandibular lymph nodes, cervical lymph nodes, salivary glands, and spleen (Figure 2B).

The following organs were examined, but no MCT was observed: lungs, heart, liver, kidneys, gastrointestinal tract, and brain.

## 3. Discussion

This case documents a malignant cutaneous MCT in a Cairo spiny mouse with local recurrence and distant metastasis. Although the splenic MCT could represent a separate primary lesion, the overall findings support metastasis from the primary cutaneous tumor. While cutaneous MCTs are well characterized in dogs and cats, they are rarely reported in rodents, particularly in nonlaboratory species.

The histologic and immunohistochemical features in this case are consistent with MCTs described in other species, including metachromatic cytoplasmic granules and positive immunostaining for c‐kit and tryptase. Using the canine criteria, the initial cutaneous MCT in this mouse would be classified as a Grade II MCT based on Patnaik′s three‐tier grading system [14] and a low‐grade MCT based on Kiupel′s two‐tier grading system [15], where the prognosis is generally favorable with complete excision [16]. Feline cutaneous MCTs do not have an established histological grading system to predict their biological behavior. Most feline cutaneous MCTs are well differentiated and are behaviorally benign, particularly those with fewer than five mitoses in 2.37 mm^2^ [2].

Neoplastic cells in this study have variably distinct basophilic to amphophilic intracytoplasmic granules, which are consistent with mast cell granules seen in cats and dogs but inconsistent with findings reported in thymic MCT in a rat, which reportedly had eosinophilic intracytoplasmic granules [9]. These granules also exhibited strong metachromasia on toluidine blue and Giemsa stains and were positive with c‐kit and tryptase immunohistochemistry [9]. While prognostic information on cutaneous MCTs in pet mice is limited in the literature, this case demonstrated aggressive biological behavior, with local recurrence and metastasis within 3 months following narrow surgical excision. Similar discordance between the benign histomorphological appearance and the malignant biological behavior has been reported in laboratory mice [5].

Native to rocky desert and shrubland areas in Africa, the Middle East, and Southern Asia, the Cairo spiny mouse (*Acomys cahirinus*) is currently classified as “least concern” by the International Union for Conservation of Nature [17]. In recent years, *Acomys* has become an emerging research species due to its unique ability (compared to other mammals) to undergo scarless tissue regeneration from skin wounds, making it a valuable research model in tissue regeneration [18]. Historically, *Acomys* has been used for Type 2 diabetes investigations, behavioral, and gestation studies [19]. In addition to a research model, certain species of spiny mice are now maintained in the exotic pet trade, where veterinary clinicians may increasingly encounter them as patients.

This case highlights several clinically relevant points. First, MCTs should be considered in the differential diagnosis of cutaneous masses in exotic rodents. Secondly, histochemical and immunohistochemical confirmation is valuable in spiny mice, similar to other species. Lastly, malignant behavior, including metastasis, can occur in cutaneous MCTs in spiny mice even when initial histologic features appear relatively low grade using canine criteria.

## 4. Conclusion

MCTs are well recognized in dogs and cats but remain rarely reported in rodents, which may lead to diagnostic challenges when evaluating cutaneous nodules in rodents. This report describes the first documented case of a cutaneous MCT in a Cairo spiny mouse and demonstrates its potential for aggressive biological behavior, including metastasis. The present case adds to the relatively small body of reports documenting neoplasia, specifically MCTs in pet mice. The outcome of this case emphasizes the importance of including MCTs in the differential diagnosis of skin masses in exotic rodent species. Increased awareness of this tumor type may aid in the diagnosis and management of cutaneous masses in exotic rodent species.

## Funding

No funding was received for this manuscript.

## Conflicts of Interest

The authors declare no conflicts of interest.

## Data Availability

The data that support the findings of this study are available from the corresponding author upon reasonable request.
